# *ZFP91*—A Newly Described Gene Potentially Involved in Prostate Pathology

**DOI:** 10.1007/s12253-013-9716-z

**Published:** 2013-11-23

**Authors:** Lukasz Paschke, Marcin Rucinski, Agnieszka Ziolkowska, Tomasz Zemleduch, Witold Malendowicz, Zbigniew Kwias, Ludwik K. Malendowicz

**Affiliations:** 1 Department of Histology and Embryology, Poznan University of Medical Sciences, 6 Swiecicki St., 60-781 Poznan, Poland; 2 Department of Urology and Urooncology, Poznan University of Medical Sciences, Poznan, Poland

**Keywords:** Benign prostate hyperplasia, Prostate cancer, Prostate cancer cell lines, ZFP91

## Abstract

In search for novel molecular targets in benign prostate hyperplasia (BPH), a PCR Array based screening of 84 genes was performed. Of those, expression of *ZFP91* (ZFP91 zinc finger protein) was notably upregulated. Limited data concerning the function of *ZFP91* product show that it is a potential transcription factor upregulated in human acute myelogenous leukemia and most recently found to be the non-canonical NF-κB pathway regulator. In order to test this finding on a larger number of samples, prostate specimens were obtained from patients undergoing adenomectomy for BPH (*n* = 21), and as a control, from patients undergoing radical cystectomy for bladder cancer (prostates unchanged pathologically, *n* = 18). Similar studies were performed on cultured human prostate cancer cell lines: LNCaP, DU145, 22Rv1, PC-3; as well as normal prostate epithelial cells—PrEC. Methods employed included: Human Obesity PCR Array (Qiagen), QPCR and Western blotting. QPCR studies confirmed significant overexpression of *ZFP91* in BPH samples. On a protein level, however, comparison between normal and BPH prostates revealed insignificant differences. As for prostate cell lines examined, all expressed *ZFP91* mRNA. Western blotting analysis showed markedly higher protein levels of ZFP91 in all cancer cell lines in comparison with normal (PrEC) cells. In conclusion, the upregulated *ZFP91* mRNA in BPH, not accompanied by parallel changes in ZFP91 protein levels, together with ZFP91 protein abundance in prostate cancer cell lines suggest *ZFP91* involvement in these prostate diseases.

## Introduction

Several lines of evidence show that prostate health could be adversely affected by obesity [1]. Among other findings, it was proven that obesity increases the risk of more aggressive prostate cancer [2], prostate enlargement in men with localized prostate cancer [3], and developing benign prostate hyperplasia (BPH) [4].

The starting point for this study was to analyze expression of obesity-related genes in BPH tissue specimens compared to control prostates. Using a PCR Array based screening of 84 genes, three genes with significantly changed expression levels were found: ZFP91 zinc finger protein (*ZFP91*), interleukin 1 receptor, type I (*IL1R1*) and tumor necrosis factor (*TNF*). However, after a conventional QPCR validation of the results on a larger number of samples, only *ZFP91* emerged as potentially involved in prostate pathology. Surprisingly enough, its presence on a “Human Obesity RT^2^ Profiler PCR Array” was a coincidence, as until yet it has no known association with obesity. Most probably it was because *ZFP91* is situated adjacent to ciliary neurotrophic factor receptor (*CNTFR*, an anorectic gene) on a 11 chromosome and a co-transcribed mRNA of the two was found in testis, however this transcript is thought to be non-coding (Gene ID: 386607).

*ZFP91* encodes a 63.4 kDa nuclear protein with structural motifs characteristic of transcription factor [5]. In mammals it is expressed ubiquitously in various cell types and is known to be highly conservative, e.g. according to NCBI human *ZFP91* shares 92.6 % homology with mouse ZFP91 at DNA level and 90.8 % at protein level [6]. In 2003, Unoki et al. found *ZFP91* expression to be markedly upregulated in mononuclear cells from patients with acute myelogenous leukemia (AML) and in many neoplastic blood cell lines. Moreover, it was shown that suppression of *ZFP91* expression could increase cell apoptosis rate [5]. To date, proteomic based studies showed ZFP91 interaction with a few proteins. Firstly, with ARF tumor suppressor (cyclin-dependent kinase inhibitor 2A, isoform 4), which is known for its induction of p53-dependent cell death or growth arrest in response to activated oncogenes [7]. Secondly, with mitogen-activated protein kinase kinase kinase 14 (MAP3K14) also known as NF-κB-inducing kinase (NIK)—a key kinase of the non-canonical (alternative) NF-κB activation pathway [8]. Lastly, with the von Hippel–Lindau tumor suppressor (pVHL) and the hypoxia inducible factor-1α (HIF-1α)—playing an important role in cancer malignancy and metastasis [9]. As for ZFP91 function, it is recognized as an atypical E3 ubiquitin-protein ligase. It mediates a K63-linked ubiquitination of MAP3K14, leading to its stabilization and activation of the non-canonical NF-κB pathway, serving as a selective regulator of this pathway target genes expression [8, 10].

In 2008 Lee et al. patented potential therapeutic methods employing ZFP91 functioning modifications. Their invention was based on several novel findings regarding ZFP91 significance. Among them, finding that *ZFP91* expression is upregulated by NF-κB binding to its promoter sequences in *ZFP91* 5′ upstream region. On the other hand, *ZFP91* overexpression results in increased NF-κB activity, which is dose dependent and dependent on MAP3K14 presence. Moreover, *ZFP91* possesses potent oncogenic properties confirmed in several experiments [9].

The present study aimed to investigate *ZFP91* expression on mRNA and protein levels in specimens from human normal prostate and BPH. Furthermore, we analyzed pattern of expression of studied mRNA and protein in prostate cancer cell lines and normal prostate epithelial cells.

## Materials and Methods

### Prostate Tissue Specimens

Studies were performed on patients from the Department of Urology and Urooncology, Poznan University of Medical Sciences. Bioethical Committee of the University provided consent for the study protocol and each participant signed an informed consent form. Prostate tissue specimens were obtained from patients undergoing adenomectomy for BPH, and as a control, from patients undergoing radical cystectomy for bladder cancer (prostate tissue unchanged pathologically). Tissue samples from control prostates were taken from the regions adjacent to the colliculus seminalis (in a preliminary study no differences in studied genes expression were found between this region and region adjacent to connective tissue capsule). BPH samples were taken from the anterior periurethral area. Material was conserved in RNALater (Ambion, Austin, USA) and kept at −20 °C until used.

### Cell Culture

Prostate cancer cell lines: LNCaP, DU145, PC-3 and 22Rv1 were obtained from American Type Culture Collection (ATCC, Manassas, USA) and maintained in RPMI-1640 Medium (LNCaP and 22Rv1), F12K Medium (PC-3) and Eagle’s Minimum Essential Medium (DU145). Media were purchased from ATCC and supplemented with 10 % fetal bovine serum and antibiotic/antimycotic solution. Normal prostate epithelial cells (PrEC) were obtained from Lonza (Walkersville, USA) and cultured in serum-free PrEGM (Lonza). The cells were grown in 75 cm^2^ flasks at 37 °C in a humidified atmosphere of 5 % CO_2_. The culture medium was changed every day for PrEC cells and every 2 days for other cell lines. When cells reached approximately 80 % confluence, they were either subcultured or harvested by 0.25 % trypsin–EDTA. Harvested cells were frozen in −80 °C for further analyses.

### Total RNA and Protein Extraction

Total RNA was extracted by means of TRI reagent, using additional Dounce homogenization for tissues, followed by a standard procedure combined with RNA purification using NucleoSpin RNA II kit (Macherey-Nagel Ltd., Oensingen, Switzerland). RNA concentration and purity was determined spectrophotometrically (NanoDrop, Thermo Scientific, Waltham, USA). For each sample 1 μg of total RNA was reversely transcribed using MMLV reverse transcriptase kit (Novazym, Poznan, Poland) using Oligo dT (PE Biosystems, Warrington, UK) as primers. The reaction was performed at 42.8 °C for 60 min (UNO II thermocycler, Biometra, Goettingen, Germany).

Total protein was extracted using homogenization in an ice-cold RIPA buffer with addition of Complete Mini Protease Inhibitor Cocktail Tablets (Roche Diagnostics Corporation, Indianapolis, USA). Afterwards, samples were incubated with an agitation for an hour and centrifuged at 20,000× g for 30 min to remove cell debris (all at 4 °C). Protein concentration was determined by Bradford method.

### Quantitative PCR Array

Human Obesity RT^2^ Profiler PCR Array was purchased from SuperArray Bioscience (SABioscience, Frederick, USA). PCR was performed on 7900HT Fast RT Real-Time PCR System (Applied Biosystems), according to the manufacturer’s instructions. For data analysis, the ΔΔCt method was used with the aid of a Microsoft Excel spreadsheet containing algorithms provided by the manufacturer. Fold-changes were then calculated and expressed as log-normalized ratios of values from BPH/control tissues.

### QPCR Analysis

**Fig. 1:**
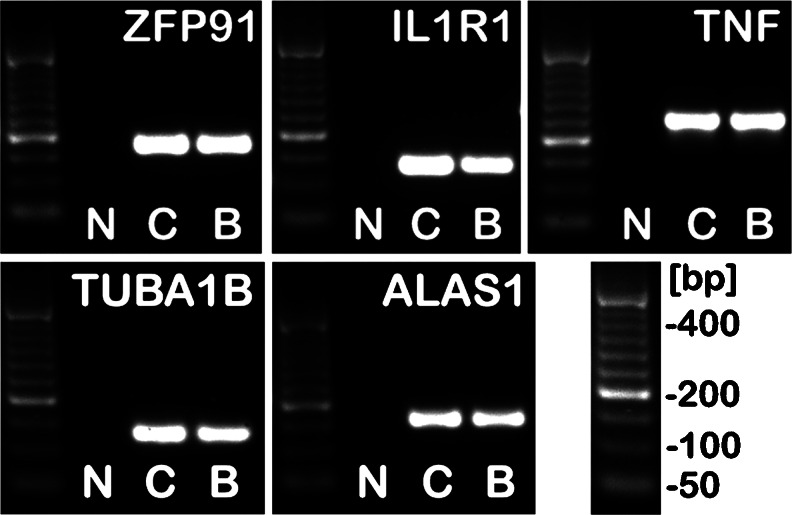
Ethidium bromide-stained 2.5 % agarose gel showing cDNA amplified with human ZFP91 zinc finger protein (ZFP91), interleukin 1 receptor, type I (IL1R1), tumor necrosis factor (TNF), tubulin alpha 1b (TUBA1B) and aminolevulinate, delta-, synthase (ALAS1) specific primers. Note presence of reaction products with the expected size: ZFP91—190 bp, IL1R1—147 bp; TNF—263 bp, TUBA1B—135 bp, ALAS1—167 bp. Lanes description: *C* control prostate, *B* BPH sample, *N* negative control (no RT of the RNA). As a DNA size standard O’RangeRuler 50 bp DNA Ladder (Fermentas) was used

Analyses were performed as described earlier [11–14]. Briefly, all primer sets were designed to span at least one intron (Table 2). They were purchased from the Laboratory of DNA Sequencing and Oligonucleotide Synthesis (Institute of Biochemistry and Biophysics, Polish Academy of Sciences, Warsaw, Poland). Real-time PCR was carried out in a Roche LightCycler 2.0 (Roche) with software version 4.05. SYBR Green detection system was used based on LightCycler FastStart DNA Master SYBR Green I mix (Roche). PCR reactions were carried out in 20 μL mixtures, containing 4 μL template cDNA, 0.2 μM of each gene-specific primer and 3.5 mM of Mg^2+^ ions. The real-time PCR program included a 10 min denaturation step to activate the Taq DNA polymerase, followed by a three-step amplification program: denaturation at 95.0 °C for 9 s, annealing at 58.0 °C for 5 s, and extension at 72.0 °C for 5 s. Specificity of the reaction products was routinely checked by determination of melting points (0.1 °C/s transition rate) and random sample separation in a 2.5 % ethidium bromide/agarose gel (Fig. 1). Templates not submitted to RT reaction served as negative controls. Out of three reference genes examined: tubulin alpha 1b (TUBA1B), aminolevulinate, delta-, synthase (ALAS1) and β2-microglobulin (B2M), two first were selected using geNorm method as a reference to normalize data. Of note, selected genes were one of 3 out of 16 proven to be the most stable and useful for normalization purposes in gene profiling studies of prostate tissues, both malignant and not [15].

PCR efficiency was assessed by a serial dilution method. Briefly, products of PCR reactions were separated in a 2.5 % agarose gel and specific bands were extracted using a DNA gel extraction kit (Millipore, Billerica, USA). The amount of extracted DNA was estimated spectrophotometrically. Extracted DNA was diluted (10-fold serial dilutions) in order to generate a standard curve for efficiency calculation (LightCycler software version 4.05.).

### Western Blot Analysis

Analyses were performed as described earlier [11–13, 16]. Briefly, for each sample, 20 μg of protein was separated in a 4–20 % gradient SDS-polyacrylamide electrophoretic gel, and transferred onto a PVDF membrane. Jurkat whole cell lysate (sc-2204; Santa Cruz Biotechnology) served as a positive control for ZFP91 detection. Transferred proteins were stained with Ponceau S. Membranes were incubated in a blocking buffer consisting of 5 % nonfat dry milk in TBST for 1 h, followed by primary antibody incubation overnight at 4 °C with rabbit anti-ZFP91 at 1:200 (sc-102172; Santa Cruz Biotechnology) or rabbit anti-GAPDH at 1:2000 (#5174; Cell Signaling Technology). Afterwards, membranes were thoroughly washed and incubated with an anti-rabbit HRP-linked antibody at 1:2000 (#7074; Cell Signaling Technology) for 1 h at room temperature. After washing, membranes were incubated with enhanced chemiluminescence (ECL Plus, Amersham) detection reagents (5 min, room temperature) and visualized on GelDoc-It Imaging System (UVP, Upland, USA) with use of VisionWorks LS Software. Band detection and quantification of band intensity was performed using TotalLab (Nonlinear dynamics, Newcastle upon Tyne, England).

### Statistical Analysis and Data Presentation

GraphPad Prism version 5.00 (GraphPad Software, San Diego, USA) was used to perform statistical analyses. All data were expressed as means ± SD. Differences were considered significant at *p* < 0.05. For a more detailed description of particular experiment statistics, see description below each figure.

## Results

### Expression of ZFP91 mRNA and Protein Levels in Benign Prostate Hyperplasia

**Fig. 2:**
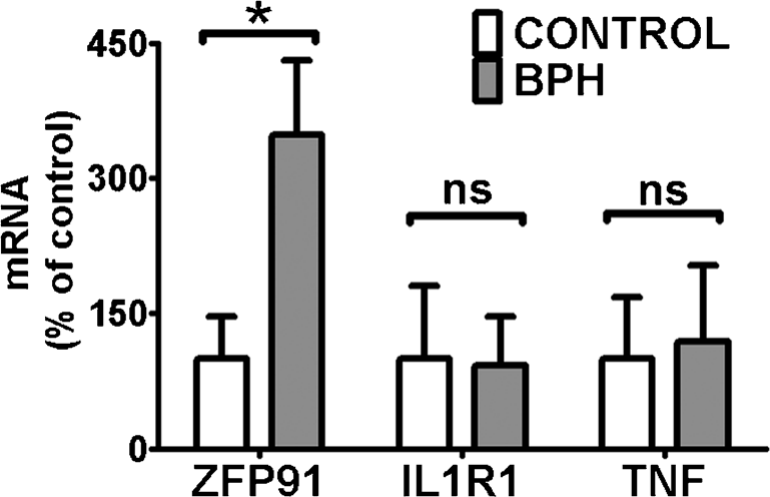
QPCR analyses of *ZFP91*, *IL1R1* and *TNF* expression in BPH (benign prostate hyperplasia) samples and control human prostates (CONTROL). *Bars* represent mRNA levels relative to reference genes. Mean expression in CONTROL was assigned a value of 100 for each gene independently. Results are presented as means ± SD; CONTROL *n* = 18, BPH *n* = 21. Statistical comparison by Mann–Whitney test; *p < 0.05, *ns* not significant

A screening of 84 obesity-related genes was performed by a quantitative PCR array (Table 1). Only three genes, namely *ZFP91*, *IL1R1* and *TNF*, had mean expression changed over threefold in BPH samples compared to control prostate tissues. Their expression was further analyzed using classic real-time QPCR (Table 2, Fig. 1) on a larger number of samples (Fig. 2). Only *ZFP91* gene expression was confirmed to be upregulated significantly in BPH specimens.

**Table 1:** Functional grouping of 84 obesity-related genes assessed by the quantitative PCR array (Human Obesity RT^2^ Profiler PCR Array; SABioscience, Frederick, MD, USA)

Orexigenic genes	
Neuropeptides and receptors:	ADRA2B, AGRP, CNR1, GAL, GALR1, MCHR1 (GPR24), HCRT, HCRTR1, NPY, NPY1R, NR3C1 (GRL), OPRK1, OPRM1.
Gut hormone and receptor:	GHRL (Ghrelin / Obestatin), GHSR.
Anorectic genes	
Neuropeptides and receptors:	ATRN (Attractin), BDNF, BRS3, CALCA, CALCR, CARTPT (CART), CNTFR, CRHR1, DRD1, DRD2, GH1, GH2, GHR, NMUR1 (GPR66), GRP, GRPR, HRH1, HTR2C, IL1A, IL1B, *IL1R1*, IL6, IL6R, MC3R, NMB, NMBR, NMU, NTRK2, NTS, NTSR1, PRLHR (GPR10), POMC, SORT1, TRH, UCN, *ZFP91*.
Gut hormones and receptors:	APOA4, CCK, CCKAR, GLP1R, PYY.
Adipocyte-derived peptides and receptors:	LEP (Leptin), LEPR, *TNF*.
Pancreas derived peptides and receptors:	CALCR, CLPS, GCG, GCGR, GLP1R, IAPP, INS, INSR, RAMP3, SST, SSTR2.
Genes related to energy expenditure	
Adipocyte-derived peptides and receptors:	ADIPOQ (ACRP30), ADIPOR1, ADIPOR2, ADRB1, C3, PPARA, PPARG, PPARGC1A, PTPN1, UCP1.
CNS-derived peptides and receptors:	ADCYAP1, ADCYAP1R1, CPD, CPE, SIGMAR1, THRB.

Genes rendered in italics had mean expression over threefold upregulated (ZFP91 and IL1R1) or downregulated (TNF) in BPH samples relative to control prostate tissues (each group *n* = 3)

**Table 2:** Conventional real-time QPCR analyses of ZFP91 zinc finger protein (ZFP91), interleukin 1 receptor, type I (IL1R1) and tumor necrosis factor (TNF) genes expression

cDNA	Genbank accession no.	Primer	Primer sequence (5′-3′)	Position	PCR product size (bp)
ZFP91	NM_053023	S	TGTCCTTGCCCATCCTCGCTA	1128–1148	190
		A	ACTCTTGAAGGCCCGAGCAC	1298–1317	
IL1R1	NM_000877	S	CCAGCTAATGAGACAATGGA	773–792	147
		A	GTAATAGTCTTCCCCTAGCA	900–919	
TNF	NM_000594	S	GCCTCTTCTCCTTCCTGATCGT	276–297	263
		A	ATCTCTCAGCTCCACGCCAT	519–538	
TUBA1B	NM_006082	S	TGGAACCCACAGTCATTGATGA	430–451	135
		A	TGATCTCCTTGCCAATGGTGTA	543–564	
ALAS1	NM_000688	S	AGACATAACATCTACGTGCAA	2031–2051	167
		A	GAATGAGGCTTCAGTTCCA	2179–2197	

Oligonucleotide sequences for sense (S) and antisense (A) primers are shown. Tubulin alpha 1b (TUBA1B) and aminolevulinate, delta-, synthase (ALAS1) served as reference genes

**Fig. 3:**
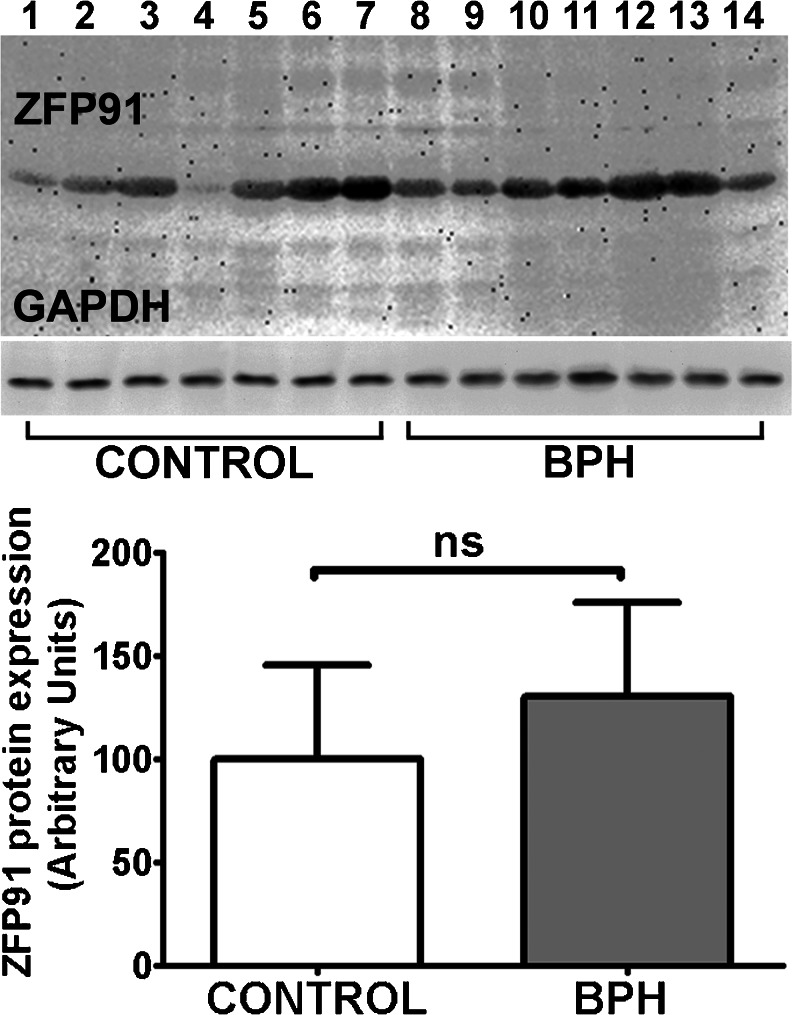
Representative experiment of ZFP91 protein immunoblotting in control prostates (CONTROL) and benign prostate hyperplasia samples (BPH). *Bars* represent protein expression relative to glyceraldehyde-3-phosphate dehydrogenase (GAPDH) levels. Results are presented as means ± SD; each group *n* = 14. Statistical comparison by Student’s t-test; *p < 0.05, *ns* not significant

In order to test this finding on a protein level, Western blotting of ZFP91 protein was performed (Fig. 3). To our knowledge this is the first study to confirm ZFP91 presence in prostate tissue and, as shown in further sections, in prostate normal and cancer cells. After quantification of ZFP91 abundance in BPH and control samples using GAPDH levels as a reference (Fig. 3), differences in ZFP91 protein levels between two groups were found to be insignificant, which stands in contrast to the mRNA levels differences.

### Expression of ZFP91 mRNA and Protein Levels in Prostate Cancer Cell Lines

**Fig. 4:**
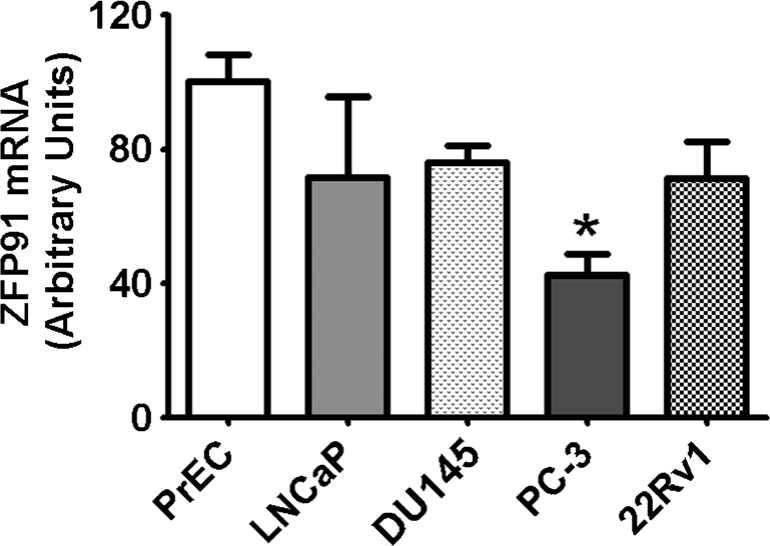
QPCR analyses of *ZFP91* expression in cultured PrEC, LNCaP, DU145, PC-3 and 22Rv1 cells. *Bars* represent mRNA levels relative to reference genes. Mean expression in PrEC cells was assigned a value of 100 and normalized in other cell lines accordingly. Results are presented as means ± SD; each group *n* = 6. Statistical comparison by ANOVA followed by Tukey’s test; *p < 0.05

QPCR analyses of *ZFP91* gene expression in cultured PrEC, LNCaP, DU145, PC-3 and 22Rv1 cells (Fig. 4) revealed stable, comparable expression in all cells studied. Normal PrEC cells had moderately higher mRNA levels than cancer PC-3 cells. However, *ZFP91* expression in both PrEC and PC-3 cells did not statistically differ from *ZFP91* expression in the rest of the cell lines studied.

**Fig. 5:**
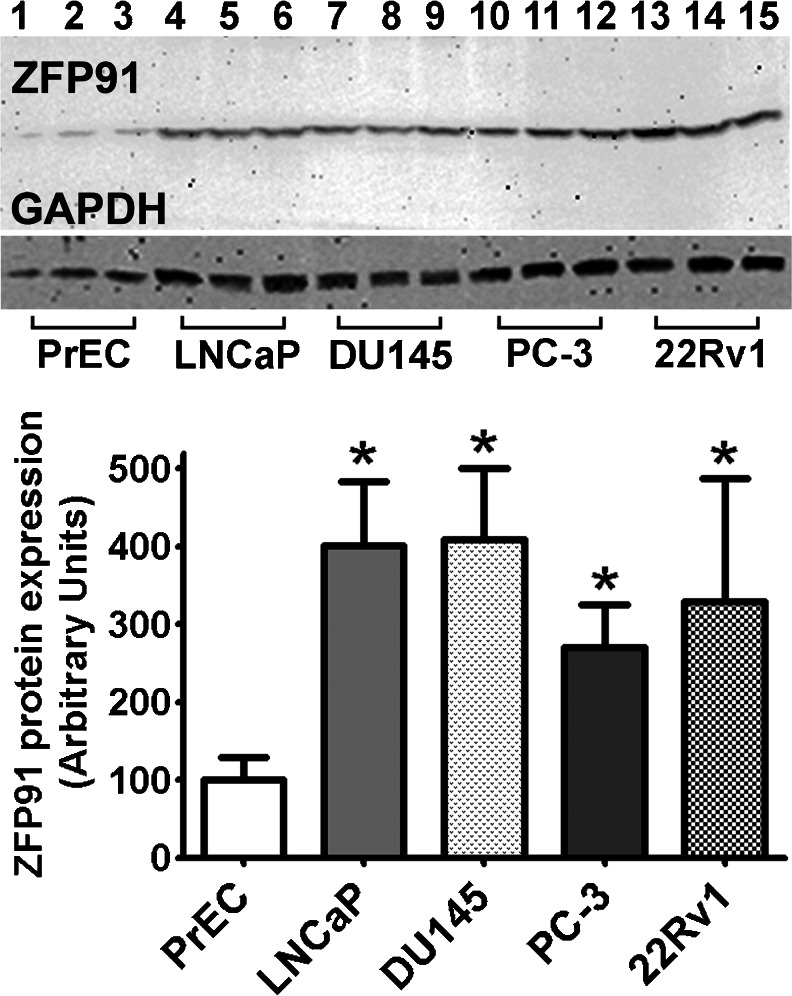
Representative experiment of ZFP91 protein immunoblotting in cultured PrEC, LNCaP, DU145, PC-3 and 22Rv1 cells. *Bars* represent protein expression relative to glyceraldehyde-3-phosphate dehydrogenase (GAPDH) levels. Mean expression in PrEC cells was assigned a value of 100 and normalized in other cell lines accordingly. Results are presented as means ± SD; each group *n* = 6. Statistical comparison by ANOVA followed by Tukey’s test; *p < 0.05

Presence of the *ZFP91* gene product in above mentioned cell lines was confirmed by means of Western blotting (Fig. 5). After quantification of ZFP91 abundance in cells using GAPDH levels as a reference (Fig. 5), markedly higher protein quantity was noted in all cancer cell lines comparing to normal prostate epithelial cells (PrEC).

## Discussion

Our study is the first to investigate ZFP91 expression at the protein level in normal and pathologically changed prostate tissues and cells. Until now, knowledge regarding *ZFP91* expression in prostate was limited to Northern blot confirmation of mRNA transcript presence in prostate [5], and finding of increased mRNA expression levels in prostate cancer specimens compared to unchanged prostate (by in situ hybridization) [9]. In our material a significant upregulation of ZFP91 mRNA was found in BPH samples, but it was not accompanied by changes in protein levels. On the other hand, prostate cancer cell lines and normal prostate epithelial cells had similar ZFP91 mRNA levels; however protein abundance was clearly the highest in cancer cell lines. This may correspond to Lee et al. 2008 observation that *ZFP91* expression induction by NF-κB pathway activators resulted in limited changes at mRNA level but significant and time-dependent at protein level.

Phenomenon of poor correlation between mRNA and protein abundance is well known in the field of gene expression analysis [17–20]. For instance, a study in lung adenocarcinomas showed that only about one fifth of investigated genes expression had significant correlation between protein and mRNA levels [21]. Although mRNA concentration is one of the important factors determining protein abundance, several other factors also influence mRNA/protein ratio. A reasonable approach to this issue is to assume that mRNA levels can be used as surrogates for corresponding protein levels, but not without verification. Results of this study show, that in case of *ZFP91* expression no clear correlation between mRNA and protein levels exist.

Taking into account ZFP91 potential function as a transcription factor and/or cell-cycle regulator, one can hypothesize that it is a protein with a fast turnover rate, subjected to rapid intracellular degradation. ZFP91 relatively high abundance in cancer cells could result from its greater stability and accumulation in these cells. Such phenomenon was observed for many oncogenic proteins, for instance p53 which abundance depends on posttranscriptional regulations [22]. Moreover, many factors in NF-κB signaling pathway are subjected to strict expression control by e.g. targeting to proteasomal degradation [23]. ZFP91 could be one of such factors in this pathway.

NF-κB is a protein complex of transcription factors participating in a range of biological processes, among them cell growth and development, cell survival and inflammatory responses. NF-κB activation results in either canonical or non-canonical (alternative) NF-κB pathway signalling, or both. Non-canonical NF-κB pathway is associated with several important biological functions including lymphoid malignancies. An important feature of this pathway is its strict dependence on MAP3K14 protein level, which is controlled mainly through its ubiquitin-dependent degradation. In this regard it should be underlined that ZFP91 serves as a positive regulator for MAP3K14, causing its stabilization and activation [24, 25]. Furthermore, oncogenic activity of the MAP3K14 protein is postulated. Its overexpression has been associated with neoplastic growth e.g. in melanoma, pancreatic carcinoma, breast cancer, lung cancer, multiple myeloma and adult T-cell leukemia. ZFP91-mediated stabilization may stand for one of mechanisms of MAP3K14 oncogenic activation [26].

In prostate cancer, clinical data and results from in vitro experiments imply that the NF-κB system is capable of interfering with androgen receptor (AR) signaling. AR interaction with NF-κB factor p52 (involved in non-canonical pathway) has a positive effect on cell growth, cell survival and AR target genes expression [27]. In the light of our studies it seems legitimate to suggest that ZFP91 functioning may influence androgen-dependent genes expression and prostate cancer biology.

Regarding *ZFP91* oncogenic potential, several experiments besides already mentioned highlight its importance. Firstly, cancer cell lines overexpressing *ZFP91* had an increased colony formation potential and invasiveness. Secondly, tumors induced in nude mice by *ZFP91* overexpressing cancer cells had overall faster growth rate and higher angiogenic potential. Lastly, *ZFP91* suppression by RNA interference resulted in decreased levels of antiapoptotic proteins and increased apoptosis rate [9]. Furthermore, ZFP91 was found to be overexpressed in malignant breast cancer and stomach cancer cell lines having high NF-κB activity in comparison with cell lines having lower NF-κB activity [9]. In studies concerning prostate pathology, NF-κB was constitutively activated in human androgen-insensitive prostate cancer cell lines such as DU145, and PC-3, but not in LNCaP androgen-sensitive cells or normal PrEC cells [28]. Our study showed however, that ZFP91 expression does not differ between DU145, PC-3 and LNCap cell lines. This suggests that above mentioned relation between basal NF-κB activity and *ZFP91* expression should not be generalized.

As far as the results of the present study are concerned, the role of upregulated *ZFP91* mRNA in BPH prostates, not accompanied by parallel changes in ZFP91 protein levels, is still to be explored. On the other hand, ZFP91 relative abundance in prostate cancer cells, together with the promising field of ZFP91 potential interactions, makes it a novel interesting target of studies. Presented work provides a base for further experiments aiming at characterizing ZFP91 role in prostate pathology.

## References

[1] Tewari R, Rajender S, Natu SM, Dalela D, Goel A, Goel MM, Tandon P (2012). Diet, obesity, and prostate health: are we missing the link?. Int J Androl.

[2] Allott EH, Masko EM, Freedland SJ (2013). Obesity and prostate cancer: weighing the evidence. Eur Urol.

[3] Kopp RP, Han M, Partin AW, Humphreys E, Freedland SJ, Parsons JK (2011). Obesity and prostate enlargement in men with localized prostate cancer. BJU Int.

[4] Parsons JK, Carter HB, Partin AW, Windham BG, Metter EJ, Ferrucci L, Landis P, Platz EA (2006). Metabolic factors associated with benign prostatic hyperplasia. J Clin Endocrinol Metab.

[5] Unoki M, Okutsu J, Nakamura Y (2003). Identification of a novel human gene, ZFP91, involved in acute myelogenous leukemia. Int J Oncol.

[6] Saotome Y, Winter CG, Hirsh D (1995). A widely expressed novel C2H2 zinc-finger protein with multiple consensus phosphorylation sites is conserved in mouse and man. Gene.

[7] Tompkins V, Hagen J, Zediak VP, Quelle DE (2006). Identification of novel ARF binding proteins by two-hybrid screening. Cell Cycle.

[8] Jin X, Jin HR, Jung HS, Lee SJ, Lee JH, Lee JJ (2010). An atypical E3 ligase zinc finger protein 91 stabilizes and activates NF-kappaB-inducing kinase via Lys63-linked ubiquitination. J Biol Chem.

[9] Lee JJ, Lee J-H, Lee K, Hong Y-S, Jin X (2008) Therapeutic agent for cancer, inflammation, and auto-immune disease containing inhibitor of Zinc Finger Protein 91. US Patent 20,080,248,024

[10] Jin HR, Jin X, Lee JJ (2010). Zinc-finger protein 91 plays a key role in LIGHT-induced activation of non-canonical NF-kappaB pathway. Biochem Biophys Res Commun.

[11] Malendowicz W, Rucinski M, Macchi C, Spinazzi R, Ziolkowska A, Nussdorfer GG, Kwias Z (2006). Leptin and leptin receptors in the prostate and seminal vesicles of the adult rat. Int J Mol Med.

[12] Paschke L, Zemleduch T, Rucinski M, Ziolkowska A, Szyszka M, Malendowicz LK (2010). Adiponectin and adiponectin receptor system in the rat adrenal gland: ontogenetic and physiologic regulation, and its involvement in regulating adrenocortical growth and steroidogenesis. Peptides.

[13] Malendowicz W, Szyszka M, Ziolkowska A, Rucinski M, Kwias Z (2011). Elevated expression of orexin receptor 2 (HCRTR2) in benign prostatic hyperplasia is accompanied by lowered serum orexin A concentrations. Int J Mol Med.

[14] Rucinski M, Ziolkowska A, Szyszka M, Hochol A, Malendowicz LK (2012). Evidence suggesting that ghrelin O-acyl transferase inhibitor acts at the hypothalamus to inhibit hypothalamo-pituitary-adrenocortical axis function in the rat. Peptides.

[15] Ohl F, Jung M, Xu C, Stephan C, Rabien A, Burkhardt M, Nitsche A, Kristiansen G, Loening SA, Radonic A, Jung K (2005). Gene expression studies in prostate cancer tissue: which reference gene should be selected for normalization?. J Mol Med (Berl).

[16] Rucinski M, Ziolkowska A, Neri G, Trejter M, Zemleduch T, Tyczewska M, Nussdorfer GG, Malendowicz LK (2007). Expression of neuromedins S and U and their receptors in the hypothalamus and endocrine glands of the rat. Int J Mol Med.

[17] Maier T, Guell M, Serrano L (2009). Correlation of mRNA and protein in complex biological samples. FEBS Lett.

[18] Taniguchi Y, Choi PJ, Li GW, Chen H, Babu M, Hearn J, Emili A, Xie XS (2010). Quantifying E. coli proteome and transcriptome with single-molecule sensitivity in single cells. Science.

[19] Maier T, Schmidt A, Guell M, Kuhner S, Gavin AC, Aebersold R, Serrano L (2011). Quantification of mRNA and protein and integration with protein turnover in a bacterium. Mol Syst Biol.

[20] Vogel C, Marcotte EM (2012). Insights into the regulation of protein abundance from proteomic and transcriptomic analyses. Nat Rev Genet.

[21] Chen G, Gharib TG, Huang CC, Taylor JM, Misek DE, Kardia SL, Giordano TJ, Iannettoni MD, Orringer MB, Hanash SM, Beer DG (2002). Discordant protein and mRNA expression in lung adenocarcinomas. Mol Cell Proteomics MCP.

[22] Koumenis C, Alarcon R, Hammond E, Sutphin P, Hoffman W, Murphy M, Derr J, Taya Y, Lowe SW, Kastan M, Giaccia A (2001). Regulation of p53 by hypoxia: dissociation of transcriptional repression and apoptosis from p53-dependent transactivation. Mol Cell Biol.

[23] Vallabhapurapu S, Karin M (2009). Regulation and function of NF-kappaB transcription factors in the immune system. Annu Rev Immunol.

[24] Harhaj EW, Dixit VM (2011). Deubiquitinases in the regulation of NF-kappaB signaling. Cell Res.

[25] Sun SC (2011). Non-canonical NF-kappaB signaling pathway. Cell Res.

[26] Xiao G, Fu J (2011). NF-kappaB and cancer: a paradigm of Yin-Yang. Am J Cancer Res.

[27] Jain G, Cronauer MV, Schrader M, Moller P, Marienfeld RB (2012). NF-kappaB signaling in prostate cancer: a promising therapeutic target?. World J Urol.

[28] Gasparian AV, Yao YJ, Kowalczyk D, Lyakh LA, Karseladze A, Slaga TJ, Budunova IV (2002). The role of IKK in constitutive activation of NF-kappaB transcription factor in prostate carcinoma cells. J Cell Sci.

